# Projecting the lives saved by continuing the historical scale-up of child and maternal health interventions in Mozambique until 2030

**DOI:** 10.7189/jogh.09.011102

**Published:** 2019-06

**Authors:** José Maiane Júnior, Réka Maulide Cane, Maria Patrícia Gonçalves, Júlia Sambo, Jacob Konikoff, Quinhas Fernandes, Kátia Ngale, Timothy Roberton

**Affiliations:** 1 Polana Caniço Health Research Center, Instituto Nacional de Saúde, Ministry of Health, Maputo, Mozambique; 2 Health Systems Cluster, Instituto Nacional de Saúde, Ministry of Health, Maputo, Mozambique; 3 Research Department, Instituto Nacional de Saúde, Ministry of Health, Maputo, Mozambique; 4 National Directorate of Public Health, Ministry of Health, Maputo, Mozambique; 5 Johns Hopkins Bloomberg School of Public Health, Baltimore, Maryland, USA

## Abstract

**Background:**

Over the past 20 years, Mozambique has achieved substantial reductions in maternal, neonatal, and child mortality. However, mortality rates are still high, and to achieve the Sustainable Development Goals (SDGs) for maternal and child health, further gains are needed. One technique that can guide policy makers to more effectively allocate health resources is to model the coverage increases and lives saved that would be achieved if trends continue as they have in the past, and under differing alternative scenarios.

**Methods:**

We used historical coverage data to project future coverage levels for 22 child and maternal interventions for 2015-2030 using a Bayesian regression model. We then used the Lives Saved Tool (LiST) to estimate the additional lives saved by the projected coverage increases, and the further child lives saved if Mozambique were to achieve universal coverage levels of selected individual interventions.

**Results:**

If historical trends continue, coverage of all interventions will increase from 2015 to 2030. As a result, 180 080 child lives (0-59 months) and 3640 maternal lives will be saved that would not be saved if coverage instead stays constant from 2015 to 2030. Most child lives will be saved by preventing malaria deaths: 40.9% of the mortality reduction will come from increased coverage of artemisinin-based compounds for malaria treatment (ACTs) and insecticide treated bednets (ITNs). Most maternal lives will be saved from increased labor and delivery management (29.4%) and clean birth practices (17.1%). The biggest opportunity to save even more lives, beyond those expected by historical trends, is to further invest in malaria treatment. If coverage of ACTs was increased to 90% in 2030, rather than the anticipated coverage of 68.4% in 2030, an additional 3456 child lives would be saved per year.

**Conclusions:**

Mozambique can expect to see continued reductions in mortality rates in the coming years, although due to population growth the absolute number of child deaths will decrease only marginally, the absolute number of maternal deaths will continue to increase, and the country will not achieve current SDG targets for either child or maternal mortality. Significant further health investments are needed to eliminate all preventable child and maternal deaths in the coming decades.

As a signatory to the Millennium Development Goals, Mozambique made important progress towards reducing maternal, neonatal, and child from 1990 to 2015. The under-five mortality rate dropped from 196 in 1997 to 97 in 2011, and the maternal mortality ratio decreased from 870 in 1997 to 408 in 2011 [1,2]. In the Mozambique Health Sector Strategic Framework (PESS) for 2014-2019, the Ministry of Health aims to “accelerate progress in reducing maternal and neonatal mortality” to achieve Sustainable Development Goals (SDGs) 3.1, “reduce the global maternal mortality ratio to less than 70 per 100 000 live births”, and 3.2, “end preventable deaths of newborns and children under 5 years of age, to reduce neonatal mortality to at least as low as 12 per 1000 live births and under-5 mortality to at least as low as 25 per 1000 live births” [3]. Mozambique is also on the verge of renewed investment in maternal and child health, through the World Bank’s Global Financing Facility. Policy makers are convening to identify the strategies that will best leverage this opportunity in the coming years [4]. If Mozambique is to achieve SDGs, judicious policy decisions will be needed to make the most of availability financial and human resources.

One technique that can guide policy makers to more effectively allocate health resources is mathematical modelling to estimate the coverage increases and lives saved that would be achieved if historical trends continue as they have in the past. With information on how coverage trends have evolved, and how they will continue, decision makers will be better positioned to choose between investment options. Projections can also be used to estimate progress towards targets, such as the SDGs, and future opportunities for improving health. Nationally representative household surveys such as the Demographic and Health Surveys (DHS) and the Multiple Indicator Cluster Surveys (MICS) are valuable resources for policy makers [5]. However, these surveys report historical data, at fixed points in time. Without additional modeling, only minimal insight can be gathered for how coverage changes will affect mortality, and specific causes of death, in the future. Such techniques are also valuable at a global level, helping to understand the opportunities and risks for countries across regions and continents. One important study that compared patterns in coverage of maternal and child interventions across countries was that by Walker et al. in 2013 [6]. The authors used logistic Loess regression models to project future coverage and contrasted countries with differing expected rates of coverage scale-up. Other studies, such as the *Generation 2030 Africa* report by UNICEF, have compared other population metrics, such as expected population growth and fertility [7].

The objective of this paper is to report estimated projections of intervention coverage from 2015 to 2030, and to show how these estimated coverage trends will affect child and maternal mortality in Mozambique. We also report the potential gains from further increasing coverage of select interventions to universal coverage levels. We hope this information will be useful to policy makers in coming years, as important decisions are made to invest in priority child and maternal health interventions.

## METHODS

### Study design

Our analysis involved four phases: (i) gathering existing intervention coverage data from historical household survey data sets; (ii) projecting future coverage trends for 2015-2030 using a Bayesian regression model; (iii) using the Lives Saved Tool (LiST) to estimate the additional lives saved by the projected coverage increases; and (iv) using LiST to estimate the further child lives saved if Mozambique were to achieve universal coverage levels of individual interventions.

### Data gathering

We gathered historical coverage data from all nationally representative household surveys conducted between 1997 and 2015 in Mozambique, including: Demographic and Health Surveys (DHS) in 1997 [1], 2003 [8], and 2011 [2]; a Multiple Indicator Cluster Survey (MICS) in 2008 [9], an AIDS Indicator Survey (AIS) in 2009 [10]; and an Immunization, Malaria, and HIV/AIDS Indicators Survey in 2015 [11]. From these data sets we selected 22 indicators for various peri-conceptual, antenatal, intra-partum, post-natal, breastfeeding, preventive, and curative interventions. We chose interventions that (a) are implemented in Mozambique, (b) have proven efficacy and known effectiveness values, (c) can be modelled in LiST, and (d) for which there is coverage data in at least two of the available data sets.

For each of the selected indicators, we determined a *start year*: the approximate year in which the health intervention was introduced in Mozambique. These *start years* were required for our projection model (discussed below) as a threshold year, after which scale-up could begin. We estimated *start years* by reviewing global and Mozambique documentation on the introduction of interventions, and by consulting experts on the interventions in the Ministry of Health.

### Projection of future coverage trends

For each indicator, we used our data to project future coverage rates for 2015-2030 using a Bayesian regression model. The core assumption of the model was that *past coverage change* predicts *future coverage change* – in other words, coverage trends will continue as they have in the past. Additional assumptions included: (1) The initial coverage increase for each indicator began no earlier than the start year, but could have begun later than the start year (in other words, the introduction of an intervention does not guarantee its immediate scale-up). (2) A change in the rate of coverage increase (a “knot”, in statistical modeling terms) occurs at some point between 1990 and 2010. The purpose of this knot was to allow for an initial steep scale-up and then a more moderate increase; or alternatively, a moderate increase that gains momentum.

The model was built as follows: For each indicator, *i* = 1, 2, …22, *j* observations *Y_ij_* were observed. Since indicators are bound between 0% to 100% we modeled log [ – 1/200 log(*Y_ij_*)] which takes on values on the entire real line. The expected mean response for the transformed variables was assumed to grow linearly at a rate of per year from the pre-defined *start year* (*sy_i_*) until the year (*η_i_*) that a knot on the trend line occurs. For times *t_ij_* greater than *η_i_* the mean transformed response was given by *α_i_t_ij_* + *β_i_* ×*α_i_*[*t_ij_* – *η_i_*] + *ϵ_i_* where *β_i_* allows for the growth rate to either slow or increase following time *η_i_*.

We estimated the trajectory of the indicators using a Bayesian mixed effects model ﬁtting:

log [ – 1/200 log(*Y_ij_*)] = *α_i_t_ij_* + *β_i_* ×*α_i_*[*t_ij_* – *η_i_*] + *ϵ_i_*

Where:

*ϵ_i_* ~ normal(0,1)

*η_i_* ~ uniform(1990 – *sy_i_*, 2010 – *sy_i_*)

*β_i_* = exp(*m_i_*) – 1, where *m_i_* ~ uniform[–log(10), log(10)]

*α_i_* = log[ – 1/200 log(*μ_i_*)] / [(2015 – *sy_i_*) + *β_i_*(2015 – *sy_i_*)], where *μ_i_* ~ uniform(0, 1)

The distribution on was interpreted as allowing slight deviations of the transformed observed values from the mean trajectory. The distribution *η_i_* makes an *a priori* assumption that the change in growth occurs between 1990 and 2010. The distribution assumes *a priori* that [*t_ij_* – *η_i_*] is equally likely to be multiplied by all values between  – 0.9*α_i_* and 9*α_i_* corresponding to various levels of decreased or increased growth. Finally, the distribution indicates *a priori* that the true indicator coverage in 2015 is equally likely to be anywhere between 0% and 100%. At *t_ij_* = 0 the response variable was forced to 0 as exp[ –200 (I_i_)] is approximately 0 for I≤3 which *a priori* should occur 100% of the time. Thus, the indicators were assumed to be zero at the *start year.*

Inference was based on 3 parallel chains ﬁt simultaneously in JAGS (version 4.2.0, 2017). Each chain consisted of 1 000 000 iterations with the ﬁrst half discarded as burn-in. The chains were thinned so that every 500^th^ iteration was kept. The Gelman-Rubin diagnostic was less than 1.05 for all model parameters indicating good convergence properties. At each iteration and for each indicator, yearly indicator coverage was calculated. The posterior mean of this coverage was taken as our best guess at the true coverage for that year. We used bootstrapped 95% confidence intervals from the 6000 retained iterations as uncertainty bounds for the yearly projection estimates.

Having estimated a future coverage rate for each indicator for each year 2015-2030, we plotted these rates as a trend. We also calculated a second trendline that vertically shifted the raw trendline such that the *projected coverage value* in the last year for which we had historical data, matched the *historical data for that year*. We calculated this second trendline before back-transforming from the modeled values, to account for the fact that all indicators must be between 0% to 100%. In calculating this shifted trendline our reasoning was that the individual year-specific historical estimates from household surveys had greater validity than the year-specific estimates projected by our model, thus it made sense for a future trendline to intersect the last known historical estimate.

### Estimation of additional lives saved and remaining deaths

Once we generated our future coverage estimates, we transferred the shifted trendline values into LiST (version 5.71, 2018). LiST is a modelling tool that uses changes in intervention coverage and known efficacy values to estimate changes in maternal, neonatal, and child mortality and the corresponding “lives saved” [12,13]. LiST can disaggregate results by intervention and cause of death, and has been used by organizations around the world for evaluation of health programs, advocacy, and priority-setting [14].

We used LiST to estimate how many additional child lives (0-59 months) and maternal lives would be saved by the projected increase in intervention coverage from baseline in 2015 until 2030, and calculated the additional lives saved attributable to each intervention. We also used LiST to estimate the deaths by cause in 2030, if historical trends in intervention coverage were to continue, and the change in maternal mortality ratio (MMR) and under-5 mortality rate (U5MR) over time. Finally, we estimated the opportunity to save further lives if Mozambique were to achieve universal coverage for individual interventions. We ran LiST analyses that scaled up each intervention individually to 90% coverage (“universal coverage”) in 2030, while keeping coverage of other interventions unchanged from our projected future trends. We then calculated the total child deaths in 2030 from all causes, and the potential additional change in child deaths resulting purely from the intensified scale-up to 90% coverage. Similar types of analysis have been conducted previously; for example, the “Missed Opportunities” analyses undertaken by the LiST team (although those analyses use a different methodology).

## RESULTS

### Future coverage trends

**Figure 1:**
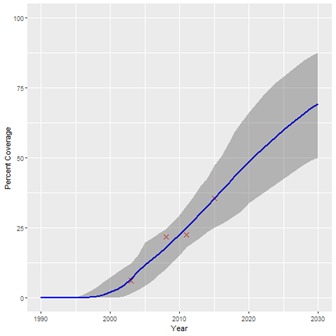
Projected coverage trend for artemisinin-based combination therapies (ACTs) for treatment of malaria. Red crosses – historical data points; Blue line – projected coverage trend; Grey shading – 95% confidence interval.

**Figure 2:**
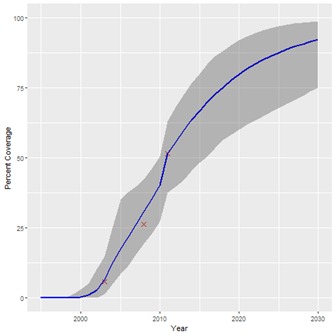
Projected coverage trend for household ownership of insecticide treated bednets (ITNs). Red crosses – historical data points; Blue line – projected coverage trend; Grey shading – 95% confidence interval.

**Figure 3:**
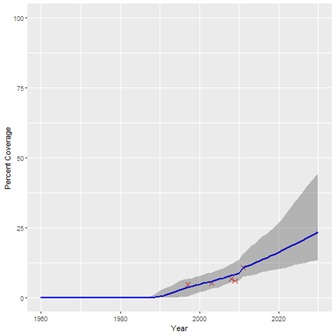
Projected coverage trend for water piped inside the household. Red crosses – historical data points; Blue line – projected coverage trend; Grey shading – 95% confidence interval.

The historical coverage data used for our projections are shown in **Table 1**, along with the start years estimated for each intervention. A summary of the estimated coverage trends calculated by our Bayesian regression model is given in **Table 2**, showing the 2030 coverage estimate for each intervention and the 95% confidence interval. **Table 2** also shows the percentage-point increase in coverage from 2015 for 2030 for each intervention. The results suggest that if historical trends continue, coverage of all interventions will increase by 2030, although not to the same degree. **Figure 1**, **Figure 2** and **Figure 3** show examples of the modelled coverage trends, with their respective uncertainty bounds. Three interventions will experience a bigger percentage-point increase from 2015 to 2030 than other interventions: artemisinin-based compounds for treatment of malaria (ACTs), increasing 32.8 percentage-points from 35.6% to 68.4%; intermittent preventive treatment of malaria in pregnancy (IPTp), increasing 30.7 percentage-points from 34.2% to 64.9%; and household ownership of insecticide treated bednets (ITNs), increasing 25.5 percentage-points from 67.5% to 92.9%.

**Table 1:** Historical coverage data and intervention start years

Intervention	Historical coverage data						Start years
**1997 (DHS)**	**2003 (DHS)**	**2008 (MICS)**	**2009 (AIS)**	**2011 (DHS)**	**2015 (AIS/MIS)**
Any breastfeeding (6-11 months)	97.5%	98.4%	97.8%		95.1%		1960
Any breastfeeding (12-23 months)	82.1%	82.7%	78.3%		75.4%		1960
Artemisinin-based combination therapies (ACTs) for malaria		6.2%	21.7%		22.5%	35.6%	1990
BCG vaccine	80.0%	90.0%	90.0%		91.0%		1945
Care-seeking for pneumonia	38.5%	55.4%	59.1%		53.6%	56.5%	1960
DPT3 vaccine	60.3%	72.7%	70.4%		77.0%	81.6%	1975
Exclusive breastfeeding (0-5 months)	30.4%	30.0%	37.2%		41.1%		1960
Facility delivery	44.3%	50.9%	58.1%		58.9%	70.3%	1960
Hepatitis B vaccine		76.0%	75.0%		76.0%		1989
Improved drinking water source	70.2%	83.2%		89.0%	84.0%		1960
Improved sanitary infrastructure	28.6%	39.4%	41.0%	41.7%	49.1%		1960
Insecticide-treated bednets (ITNs)		5.8%	26.2%		51.5%		1995
Intermittent preventive treatment of malaria in pregnancy (IPTp)			44.1%	40.7%	20.4%	34.2%	1995
Measles vaccine	57.8%	76.8%	65.5%		81.6%	82.7%	1975
Oral rehydration solution (ORS) for diarrhea	41.9%	48.7%	38.2%		55.1%	45.9%	1980
Prenatal care (PNC): 4+ visits	40.8%	53.0%			48.4%	54.6%	1960
Polio vaccine	58.0%	67.0%	74.0%		73.0%		1965
Safe disposal of child’s faeces		57.5%	56.9%		77.8%		1960
Skilled birth attendant	44.6%	49.9%	55.3%		56.0%	73.0%	1960
Tetanus vaccine in pregnancy: 2+ doses	30.9%	58.7%	66.8%		66.3%		1965
Vitamin A (for children 6-59 months)		52.0%	72.0%		75.2%		1990
Water piped inside the household	4.9%	5.3%	6.9%	6.0%	10.8%		1960

DHS – Demographic and Health Surveys, MICS – multiple indicator cluster survey, AIS -

**Table 2:** Summary of estimated coverage trends

Intervention	2015 coverage (historical observation or projected estimate)	2030 coverage (projected estimate) and 95% confidence interval	Percentage point (pp) increase from 2015 to 2030
Any breastfeeding (6-11 months)	95.7%*	97.2% (96.1%-98.4%)	1.6 (0.5-2.7)
Any breastfeeding (12-23 months)	77.5%*	83.9% (78.3%-90.5%)	6.4 (0.8-13)
Artemisinin-based combination therapies (ACTs) for malaria	35.6%	68.4% (48.5%-86.7%)	32.8 (12.9-51.1)
BCG vaccine	91.9%*	94.4% (91.9%-97.7%)	2.5 (0-5.8)
Care-seeking for pneumonia	56.5%	67.1% (58.3%-77.9%)	10.6 (1.8-21.4)
DPT3 vaccine	81.6%	89.8% (85.8%-94.3%)	8.2 (4.2-12.7)
Exclusive breastfeeding (0-5 months)	44.7%*	57.3% (45.2%-74.5%)	12.6 (0.4-29.7)
Facility delivery	70.3%	81.4% (74.1%-89.4%)	11.1 (3.8-19.1)
Hepatitis B vaccine	80.9%*	92.1% (87.1%-97.6%)	11.2 (6.2-16.7)
Improved drinking water source	86%*	91.3% (86.4%-97%)	5.3 (0.4-11)
Improved sanitary infrastructure	53.3%*	66.7% (55.8%-84.2%)	13.4 (2.5-30.9)
Insecticide-treated bednets (ITNs)	67.5%*	92.9% (78.8%-99.2%)	25.5 (11.3-31.7)
Intermittent preventive treatment of malaria in pregnancy (IPTp)	34.2%	64.9% (48.4%-85.8%)	30.7 (14.2-51.6)
Measles vaccine	82.7%	90.9% (86.2%-95.7%)	8.2 (3.5-13)
Oral rehydration solution (ORS) for diarrhea	45.9%	65.7% (54.3%-78%)	19.8 (8.4-32.1)
Prenatal care (PNC): 4+ visits	54.6%	65% (55.7%-76.8%)	10.4 (1.1-22.2)
Polio vaccine	76.1%*	85% (77.3%-92.5%)	8.9 (1.2-16.4)
Safe disposal of child’s faeces	81.3%*	89.5% (78%-98.5%)	8.2 (-3.4-17.2)
Skilled birth attendant	73%	83.4% (72.9%-91.3%)	10.4 (-0.1-18.3)
Tetanus vaccine in pregnancy: 2+ doses	70%*	80.6% (71.2%-91.8%)	10.6 (1.2-21.8)
Vitamin A (for children 6-59 months)	81.6%*	93.9% (86.8%-98.4%)	12.3 (5.2-16.8)
Water piped inside the household	13.3%*	24.5% (13.5%-49.3%)	11.2 (0.2-36.1)

*Projected estimate.

Interventions with the lowest baseline coverage (less than 40%) are generally expected to increase at a greater rate than the interventions with median (41%-70%) and higher baseline coverage (71%-100%). This makes sense as coverage gains may be more difficult to achieve for interventions that already have high coverage; further increases require coverage of harder-to-reach populations. For this reason, coverage of vaccinations will increase more slowly than other interventions, with most childhood vaccines (BCG, DPT3, measles, polio) increasing by fewer than 9 percentage-points from 2015 to 2030.

### Lives saved by continuing historical coverage trends

**Table 3** and **Table 4** provide the results of our LiST analyses, showing the additional child and maternal lives saved cumulatively from 2015 to 2030 for each intervention, and the proportion of additional lives saved contributed by each intervention among all interventions. We estimate that 180 080 additional child lives (0-59 months) and 3640 additional maternal lives will be saved by the continued increase in coverage of the 22 modelled interventions, beyond those lives already being saved at baseline (2015). Over one third (40.9%) of the additional child lives saved will be saved by two malaria-related interventions: ACTs (24.5%) and ITNs (16.4%). Labor and delivery management is estimated to contribute the most among maternal interventions, responsible for 29.4% of the additional maternal lives saved.

**Table 3:** Additional child lives saved (0-59 months), 2015-2030, by intervention

Intervention	Projected additional child lives saved 2015-2030	Proportion of total
Artemisinin-based combination therapies (ACTs) for malaria	43 869	24.5%
Insecticide-treated bednets (ITNs)	29 302	16.4%
Age-appropriate breastfeeding practices	18 661	10.4%
Oral rehydration solution (ORS) for diarrhea	16 219	9.1%
Case management of neonatal sepsis/pneumonia	11 043	6.2%
Labor and delivery management*	10,631	5.9%
Oral antibiotics for pneumonia	10 420	5.8%
Pneumococcal vaccine	6989	3.9%
Water connection in the home	6064	3.4%
Improved water source and improved sanitation	4432	2.5%
Neonatal resuscitation	4,375	2.4%
Vitamin A supplementation	2,522	1.4%
Case management of premature babies	2249	1.3%
Clean birth practices	2186	1.2%
Immediate assessment and stimulation	1809	1.0%
Intermittent preventive treatment of malaria in pregnancy (IPTp)	1771	1.0%
DPT vaccine	1696	0.9%
Hygienic disposal of children’s stools	1621	0.9%
ART	1029	0.6%
Antibiotics for pPRoM	806	0.5%
PMTCT – prevention of mother to child transmission of HIV (including breastfeeding choices)	721	0.4%
Tetanus vaccine in pregnancy: 2+ doses	644	0.4%
Measles vaccine	526	0.3%
Vitamin A for treatment of measles	373	0.2%
Maternal age and birth order	65	0.0%
Cotrimoxazole	57	0.0%
**Total**	**180** **080**	**100.0%**

*Including assisted vaginal delivery, manual removal of the placenta, caesarean section.

**Table 4:** Additional maternal lives saved, 2015-2030, by intervention

Intervention	Projected additional maternal lives saved 2015-2030	Proportion of total
Labor and delivery management*	1070	29.4%
Clean birth practices	621	17.1%
Active management of the third stage of labor	541	14.9%
Contraceptive use	480	13.2%
MgSO4 management of eclampsia	360	9.9%
Antibiotics for pPRoM	221	6.1%
Intermittent preventive treatment of malaria in pregnancy (IPTp)	174	4.8%
Insecticide-treated bednets (ITNs)	125	3.4%
Tetanus vaccine in pregnancy: 2+ doses	48	1.3%
**Total**	**3640**	**100.0%**

pPRoM – preterm premature rupture of the membranes, MgSO_4_ – magnesium sulfate

*Including assisted vaginal delivery, manual removal of the placenta, caesarean section.

**Figure 4:**
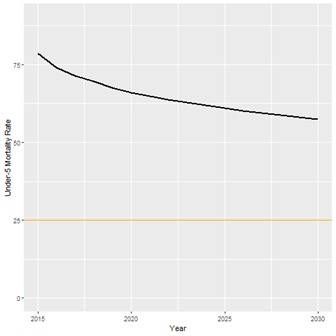
Projected trend for Under-5 Mortality Rate (U5MR). Black line – projected trend for Mozambique U5MR; Orange line – SDG 3.2 target (25 under-5 deaths per 1000 live births).

**Figure 5:**
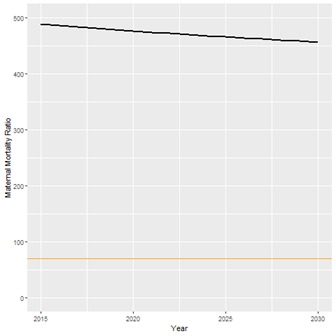
Projected trend for Maternal Mortality Ratio (MMR). Black line – projected trend for Mozambique MMR; Orange line – SDG 3.1 target (70 maternal deaths per 100 000 live births).

We also calculated the estimated change in the number of child and maternal deaths from 2015 to 2030. Because of increases in intervention coverage, the number of child deaths per year from malaria and diarrhea will decrease by 4967 and 1954, respectively. However, because of population growth, the absolute number of child deaths due to other causes will only decrease from 83 458 to 80 140, and the absolute number of maternal deaths will increase from 5300 to 6468. The only cause of maternal death that is expected to see fewer deaths is abortion, decreasing from 164 deaths per year to 149. Assuming historical trends continue, the causes of child deaths that are expected to contribute the most deaths in 2030 are neonatal prematurity (10,868), child pneumonia (9,660), and neonatal asphyxia (8,718). Most maternal deaths in 2030 are expected to be caused by indirect causes of death (3796 out of 6468 total maternal deaths), which include obstructed labor, maternal malaria, and HIV. **Figure 4** and **Figure 5** show the LiST estimates for the trend in under-5 mortality rate (U5MR) and maternal mortality ratio (MMR), because of the projected changes in intervention coverage.

### Opportunities to save further child lives by achieving universal coverage levels

**Table 5** gives the results of our final analysis, showing the opportunity to save further child lives if Mozambique were to achieve 90% coverage for each intervention. These figures show the total child deaths from all causes in 2030 if select interventions were scaled-up, while other interventions continue to increase per the historical trends in **Table 2**. The right-most column in **Table 5** shows the potential further reduction in child deaths per year for each targeted intervention. As can be seen, scaling-up all ACTs to 90% would prevent 3456 additional child deaths, even though ACTs is already expected to contribute the most additional child lives saved from 2015-2030.

**Table 5:** Potential gains by scaling interventions further to 90% coverage

Individual intervention scaled up to 90% coverage	Total child deaths in 2030 from all causes	Potential additional change in child deaths per year
**No additional scaling (2030 current expected)**	**80 140**	**–**
Artemisinin-based combination therapies (ACTs) for malaria	76 684	-3456
Facility delivery	77 148	-2992
Care-seeking for pneumonia	77 239	-2901
Oral rehydration solution (ORS) for diarrhea	77 726	-2414
Exclusive breastfeeding (0-5 months)	78 273	-1867

## DISCUSSION

The findings of our analysis suggest some promising signs for population health in Mozambique. If decision makers continue to invest in health service delivery as they have in past decades, coverage of the child and maternal interventions analysed in this paper will increase from 2015 to 2030, and as a result, 180 080 child lives (0-59 months) and 3640 maternal lives will be saved that would not be saved if coverage instead stays constant. The biggest expected gains relate to childhood malaria, with 40.9% of the estimated reduction in child mortality to come from increased coverage of ACTs and ITNs, if historical trends continue. Despite this, the biggest opportunity to save even further child lives is to additionally increase the coverage of ACTs to universal coverage levels, from an anticipated coverage of 68.4% in 2030, to an ideal coverage of 90% or beyond.

Our analysis also highlights the flat past and future trends in maternal mortality compared to child mortality. The proportion of women delivery at facilities is expected to increase from 70.3% to 81.4%, driving gains in the number of maternal lives saved from labor and delivery management. However, despite the increased number of women delivering at facilities, the absolute number of maternal deaths per year is expected to *increase*, because of population growth. LiST estimates of maternal mortality have limitations (discussed below), but even so, these results suggest the need for significant investment in maternal health. If trends continue as they have in the past, the absolute number of maternal deaths in Mozambique will increase in the future, not decrease. Furthermore, if historical trends continue, Mozambique is unlikely to achieve either SDG 3.1 or 3.2.

Aspects of our analysis echo the findings from Walker et al. in their comparable study on changes in coverage of interventions [6]. That study also estimated a relatively slow decline in mortality (“less than 28% by the year 2035 relative to 2010”) for most countries, including Mozambique, and that “continuing past trends in coverage change will not be sufficient for most countries to reach the target of an U5MR of 20 by 2035” [6]. Walker et al. also estimated that antimalarial treatment, oral rehydration solutions (ORS), and care-seeking for pneumonia would not see substantial scale-up and would remain opportunities for improvement in 2035 (our estimates have each of these interventions at 65%-69% in 2030).

These estimates are only a starting point to demonstrate that Mozambique has opportunities to further reduce child and maternal mortality, and to show that some interventions and causes of death are better positioned for improvement than others. Our study proposes a methodology to estimate future coverage of interventions based on historical trends. The strengths of this methodology are that it considers country-specific historical data to compute future trends (rather than relying on regional or global estimates); and that it incorporates the comprehensive approach to mortality modeling inherent in LiST, calculating the comparable impact of all 22 interventions at the same time, with evidenced-based effectiveness values for each intervention from the scientific literature. Projections such as these can help to make better decisions when planning policy for upcoming years and for deciding between investment possibilities. However, for truly robust policy-making, these projections should be accompanied by additional analyses on implementation challenges, costs, models of delivery, feasibility, and sustainability.

### Limitations

The 2015-2030 trends projected by our analysis assume that coverage will increase at the same rate in the future as it has historically. Although this is a straightforward assumption that is relatively simple to understand, the fact is that coverage is unlikely to increase in the future exactly as it has in the past; certainly not without policy investments at similar or higher levels than previously. The risk involved in developing such projections is that recipients will interpret the trends as a natural, secular progression – an inevitable improvement over time. The truth, however, is that any such trends will require continued direct and indirect investment in the health system, and arguably *even more* investment than previous years, as improving coverage will require reaching harder-to-reach populations.

Our projections are also highly dependent on the available data. Mozambique has been implementing MNCH interventions since 1945, but there is limited data on these interventions until 1997, when the first DHS in Mozambique was conducted. Because our future projections are a function of historical data, any inaccuracy in the historical data will be reflected in the projections. Furthermore, our analysis is limited by the exclusion of interventions for which there is not any available data. As such, the mortality reductions estimated here likely underestimate the true mortality reductions if all relevant health interventions and known risk factors were considered.

Finally, our estimates of lives saved reflect the limitations inherent in LiST. As a deterministic model for estimating mortality changes, LiST is a well-supported and popular tool [14]. However, as with any model, LiST estimates are only as accurate as the data used for the model, and some default data within LiST are more speculative than others. Notably, LiST’s effectiveness values for childbirth interventions, used to model maternal and neonatal mortality, are based on Dephi estimates, not empirical observations. Also, due to limitations in data collection methods for childbirth interventions, LiST uses coverage of “institutional delivery” as a proxy for coverage of specific childbirth interventions (such as labor and delivery management, clean childbirth practices, neonatal resuscitation), which introduces further uncertainty. For this reason, the estimates of maternal lives saved reported in this paper may be different if we had more robust data for the effectiveness and coverage of childbirth interventions in Mozambique.

## CONCLUSIONS

If historical trends continue in Mozambique, 180 080 child lives (0-59 months) and 3640 maternal lives will be saved that would not be saved if coverage instead stays constant from 2015 to 2030. Most child lives will be saved by increased coverage of ACTs, ITNs and age-appropriate breastfeeding, and most mothers by increased labor and delivery management and clean birth practices. However, even with the projected coverage increases, the number of child deaths per year will decrease only marginally, and the number of maternal deaths will increase, due to population growth outpacing coverage improvements. Fewer children will die per year from malaria and diarrhea, but *more* children will die per year from other causes of death, such as neonatal prematurity, neonatal asphyxia, and injury. Mozambique will achieve neither SDG 3.1, nor 3.2. As Mozambique strives to eliminate preventable child deaths in the coming decades, the most rapid gains could come from further increasing coverage of ACTs, oral antibiotics for pneumonia, and ORS, and by increasing facility deliveries.
